# Supplementary material for: Multimodal, label-free fluorescence and Raman imaging of amyloid deposits in snap-frozen Alzheimer’s disease human brain tissue
Source: Commun Biol. 2021 Apr 15;4:474. doi: 10.1038/s42003-021-01981-x (PMC8050064; doi:10.1038/s42003-021-01981-x)
Supplement: Supplementary file 2 — Supplementary Information [file 42003_2021_1981_MOESM2_ESM.pdf]

Supplementary Information:

Multimodal, label-free fluorescence and Raman imaging of amyloid deposits in snap-frozen Alzheimer's disease human brain tissue

Benjamin Lochocki[1], Baayla D. C. Boon[2], Sander R. Verheul[1], Liron Zada[1], Jeroen J. M. Hoozemans[2], Freek Ariese[1], Johannes F. de Boer[1]

1) Department of Physics and Astronomy, LaserLaB Amsterdam, VU Amsterdam, The Netherlands
2) Department of Pathology, Amsterdam Neuroscience, Amsterdam UMC – location VUmc, Amsterdam, The Netherlands

Plaque / control	#1a	#1b	#2	#3a	#3b	#3c	#4a	#4b	#4c	#5	c#1a	c#1b	c#1c	c#2a	c#2b
Auto-fluorescence	●	●	●	●	●	●	●	●	●	●	●	●	●	●	●
Pre-Resonance Raman	●	●	●	●	●	●	●	●	●	●	●	●	●	●	●
SRS	●	●	●	●	●	○	●	●	○	●	●	○	●	●	●
Thio-S fluorescence	●	●	●	●	●	●	●	●	●	●	●	●	●	●	●

Table ST1: Overview of imaging modalities applied to each plaque/control (see Table 1). All AD and control sections were imaged using auto-fluorescence, pre-resonance Raman and subsequent fluorescence imaging after Thioflavin-S staining. All cases were images using SRS, except AD cases #3c and #4c, and control case c#1b. ● available, ○ not available.

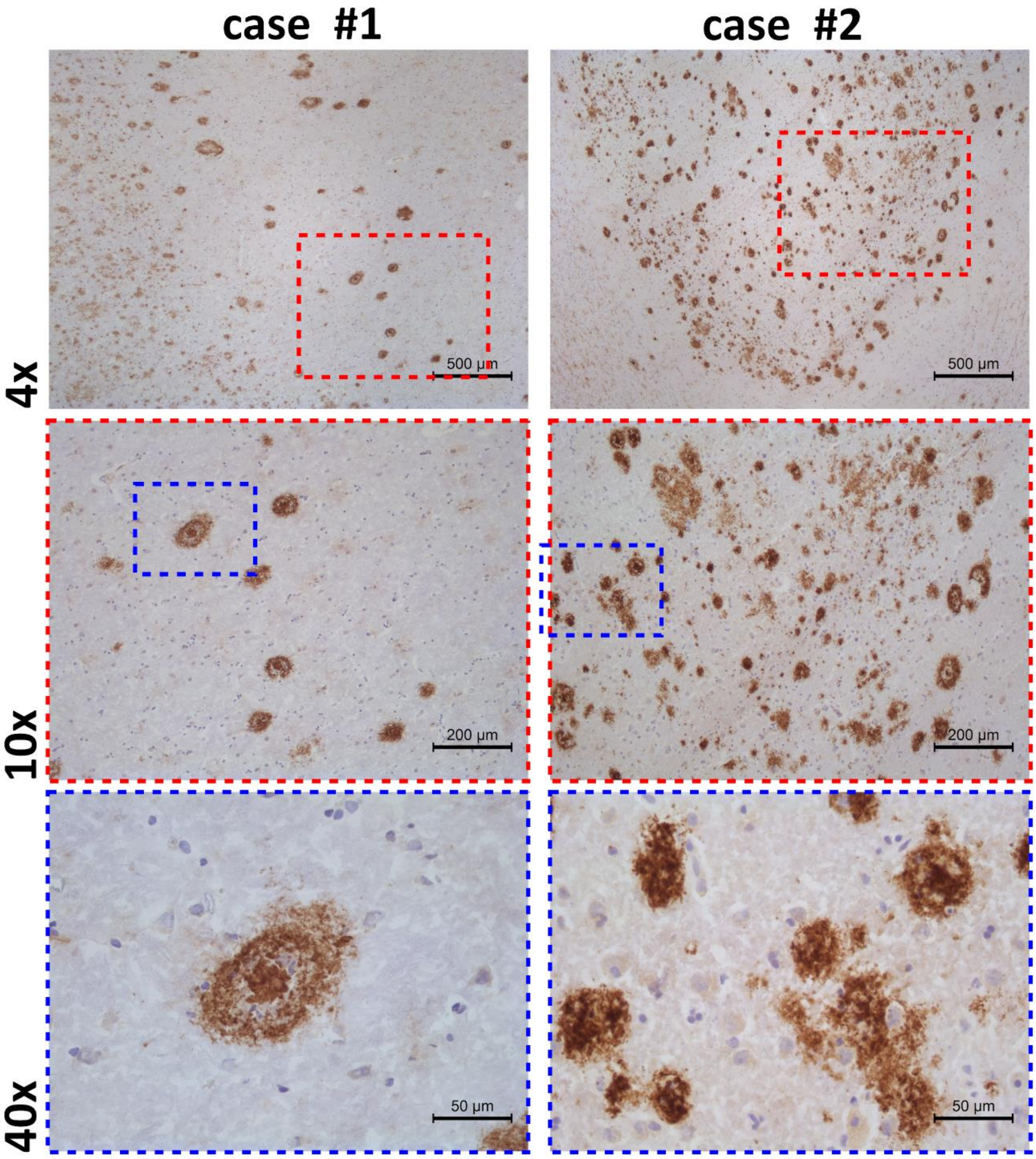

Figure S1: Amyloid-β immunostainings of nearby sections of case #1 and #2, stained with IC-16. The sections are not directly adjacent to the sections presented in the manuscript but are shown here as a visual impression of the number and type of amyloid-beta plaques present in the tissue. Case #1 shows a clear presence of classic cored plaques whereas case #2 has mainly fibrillary amyloid-beta deposits. Similar images were obtained for the other AD cases (not shown).

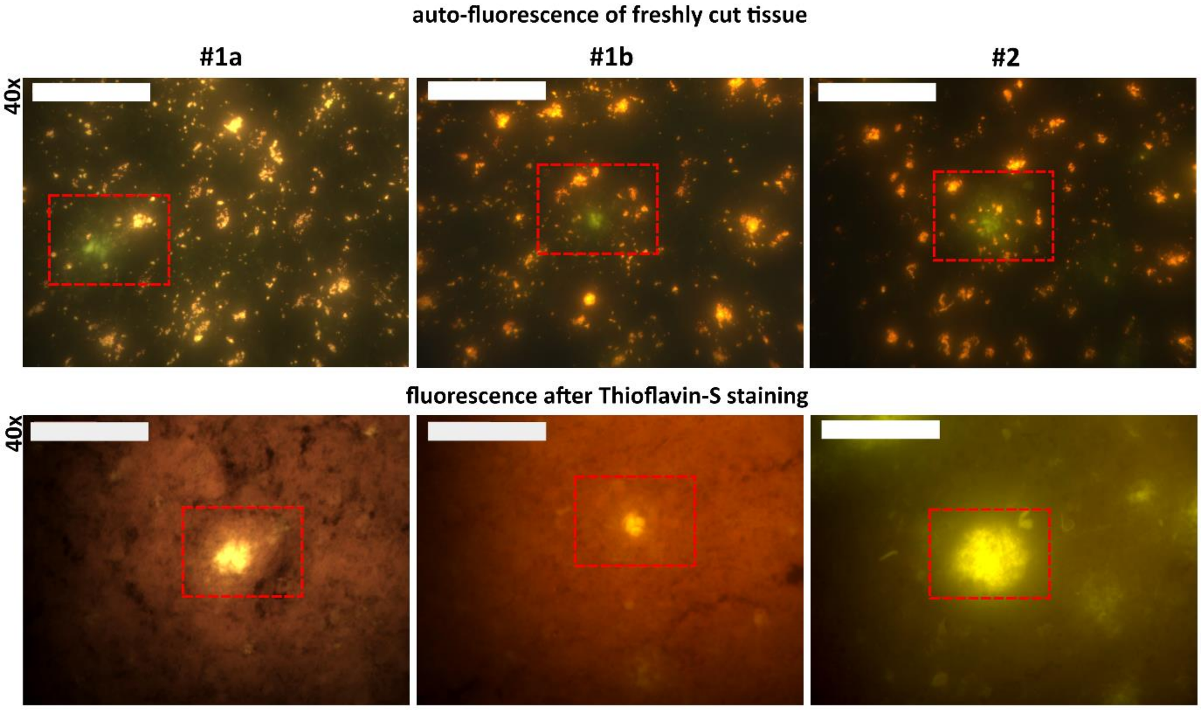

Figure S2: Additional examples of fluorescence images before and after staining. Top row: Auto-fluorescence images of unstained tissue. Red boxes highlight areas with greenish auto-fluorescence (see also Figure 2 in manuscript). Bottom row: The same tissue after Thioflavin-S staining, confirming that areas which appeared green were indeed amyloid plaques. All images taken with a 40x objective using the same microscope and a 470 nm illumination source. Scale bar 100 µm.

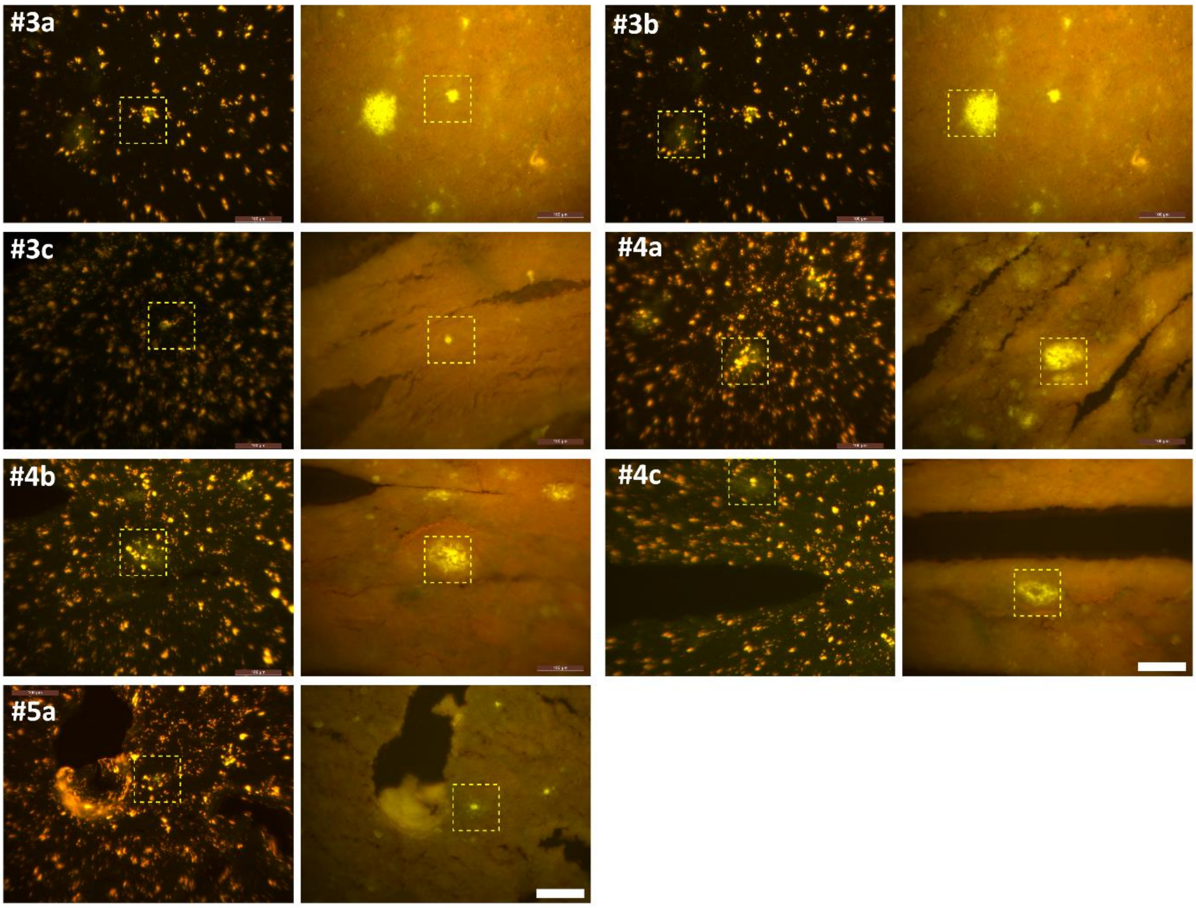

Figure S3: Fluorescence images of AD cases #3 to #5 before and after staining. Yellow dashed boxes mark roughly the same area (just for viewing purpose) Detailed areas can be seen in Figure 3. Scale bar: 100µm (valid for all images)

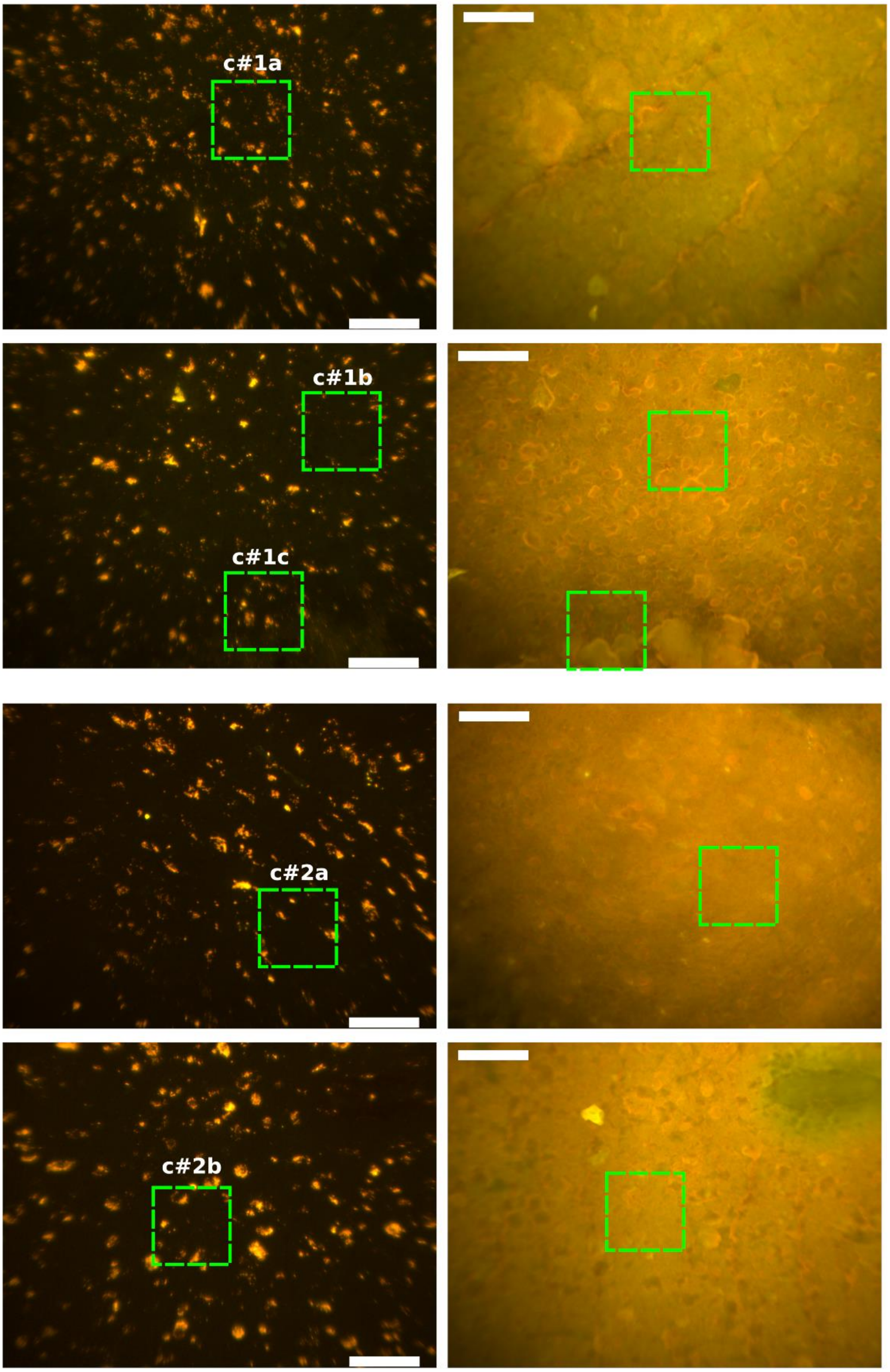

Figure S4: Fluorescence images of all control cases before and after staining, showing the absence of green emission in the unstained slices (left panels). The green dashed boxed mark the area where all the image modalities were performed. Scale bars: 100 µm. Please note that lipofuscin is also found in the control tissue and therefore supports the claim that lipofuscin deposits are not a hallmark to identify AD.

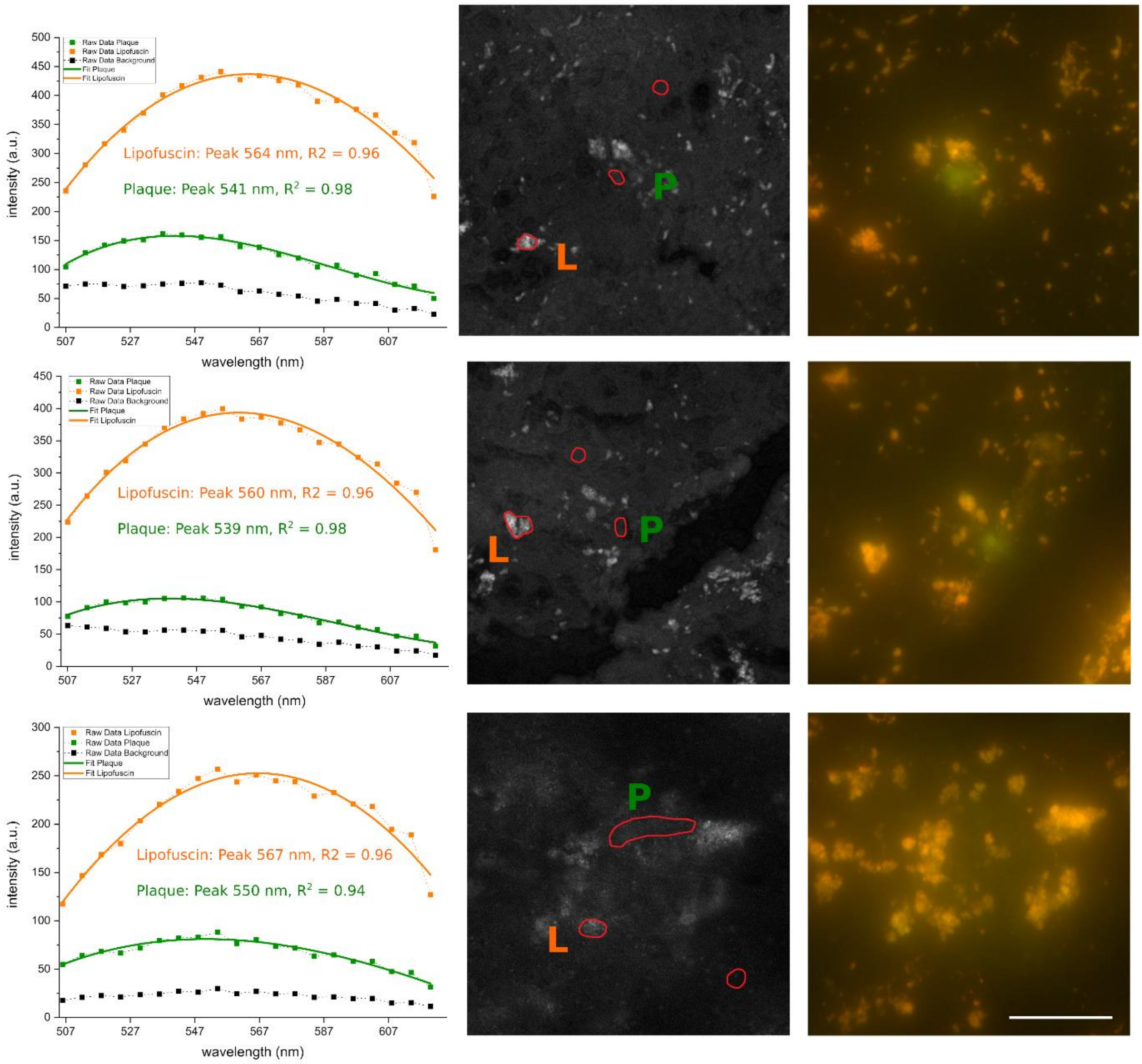

Figure S5: Fluorescence emission spectra of two dense core amyloid deposits (top rows, adjacent slice of case #3) and one fibrillar amyloid deposit (bottom row, adjacent slice of case #4) when excited with 488 nm. Symbols connected with a dashed line are the measured data. The solid lines are the corresponding 3rd order polynomial fits; peak position and R^2 values are indicated. The right half shows the corresponding z-projection of the emission images and the locations where the data points for plaque (P), lipofuscin (L) and background were taken, next to it the corresponding full-field auto-fluorescence images. Corresponding thioflavin-S images are not shown. Scale bar: 40 µm.

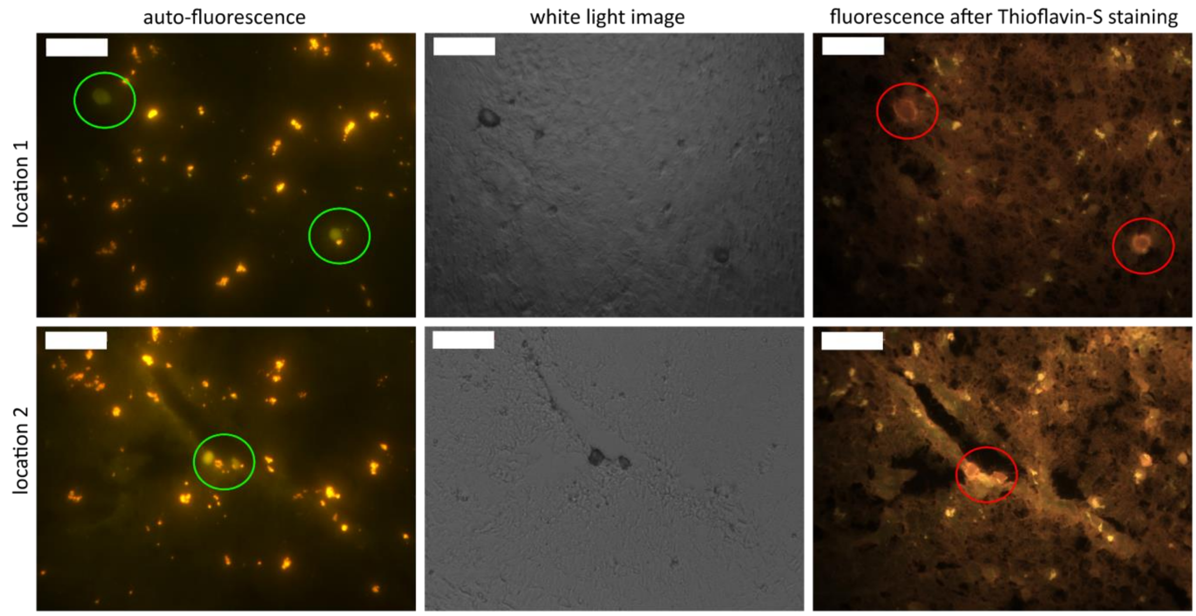

Figure S6: Example of two locations within the same tissue section (adjacent slice of #1a) with similar greenish auto-fluorescence (left column). Compared to plaques they are homogenously filled and geometrically shaped and similar to Corpora Amylacea. Middle column: white light images; right column: after staining with Thioflavin-S, these spots do not show bright yellow emission. Scale bar: 50 µm.

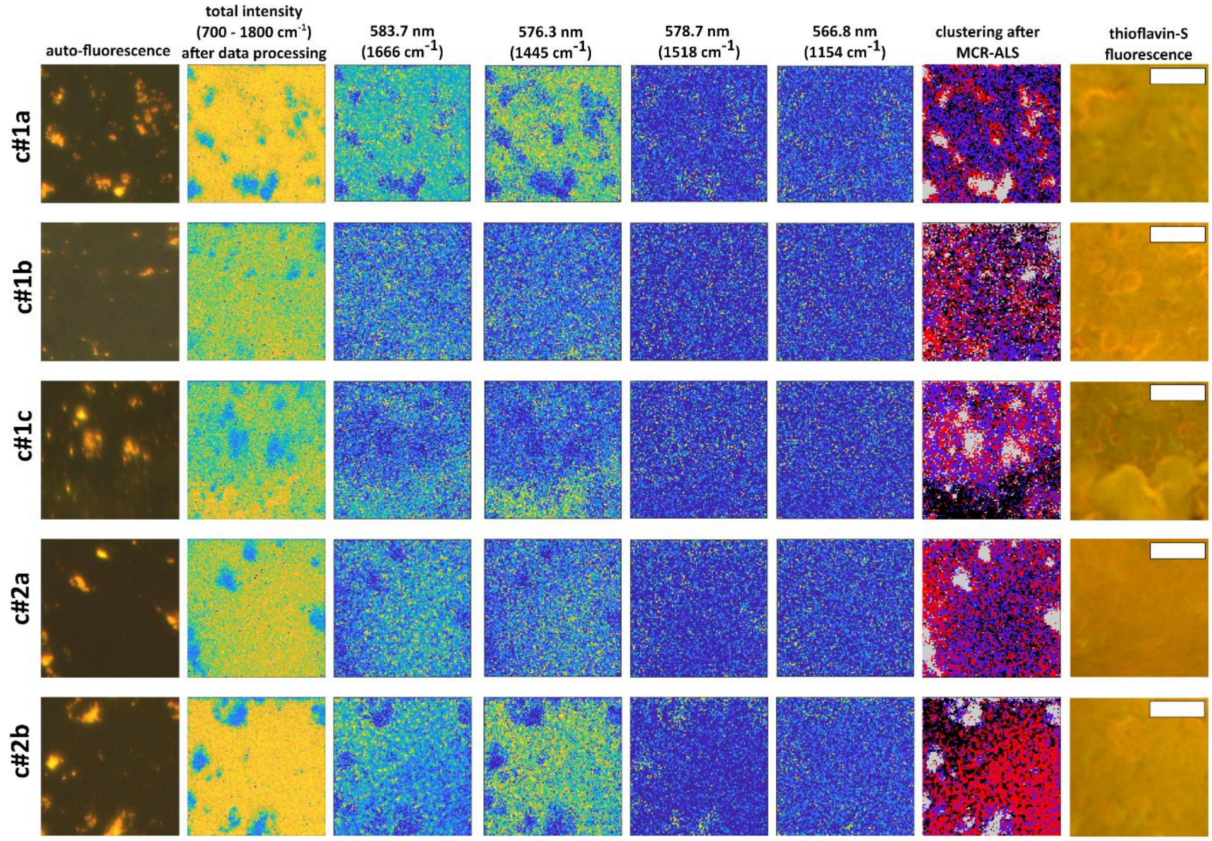

Figure S7: Overview of fluorescence and Raman images of the control cases (101 x 101 µm); each row represents one tissue section. 1st column: Auto-fluorescence images. 2nd column: The total intensity image of the spectral Raman data after data pre-processing. 3rd and 4th column: Raman peak intensity images of the protein (1666 cm^{-1}) and lipid (1445 cm^{-1}) bands. 5th and 6th column: Raman peak intensity images at two prominent carotenoid wavenumbers (1518 cm^{-1} and 1154 cm^{-1}). 7th column: 4-cluster images of the Raman data after MCR-ALS computing, highlighting lipofuscin spots in gray. Last column (8th): Fluorescence image of the thioflavin-S stained tissue, confirming the absence of plaque in these tissues. Scale bars: 40 µm.

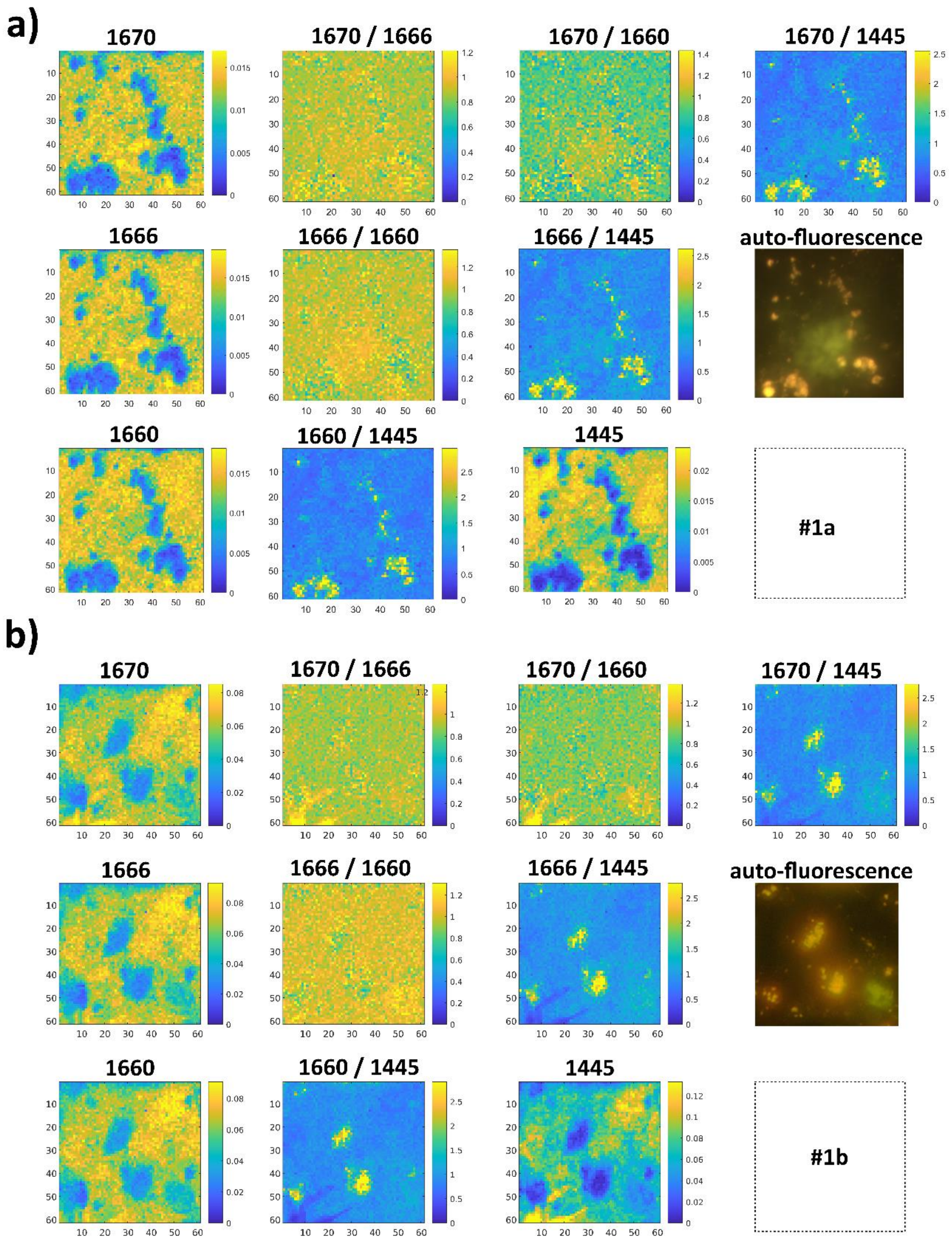

Figure S8: Raman peak intensity ratio images of cases #1a (upper half) and #1b (lower half). Images computed after smoothing, baseline removal and normalization (as described in the data processing paragraph). For the other AD cases we observe similar images but these are not shown here.

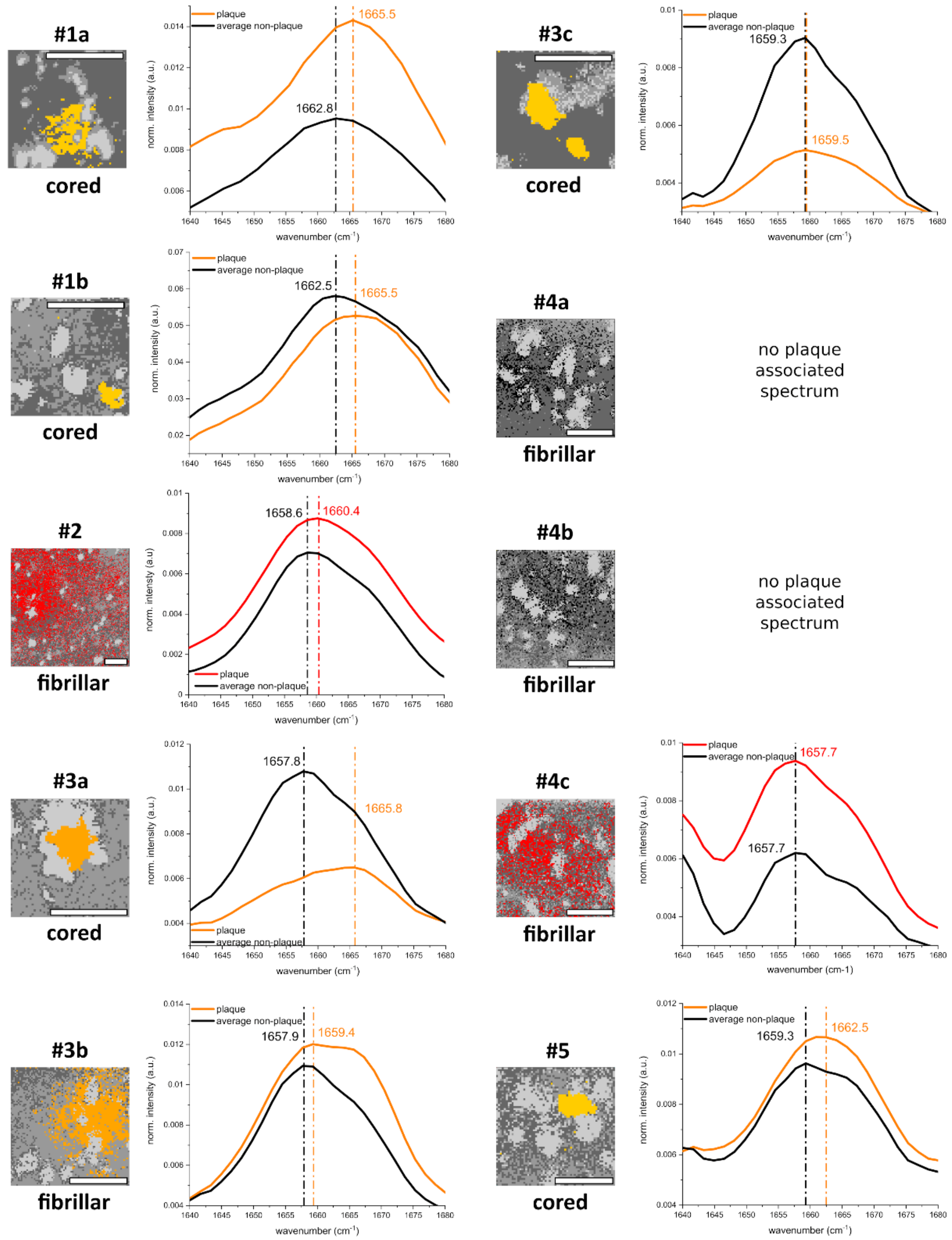

Figure S9: Spontaneous Raman Amide-I band comparison of each AD case of the plaque spectrum (orange/red line) to the averaged non-plaque spectrum (black line) based on the spectra obtained after the MCR-ALS cluster analysis. The cored plaque cases (and case #3b, which we defined as fibrillar) exhibit in general a small shift towards higher wavenumbers. For the fibrillar cases #2 and #4c the results are not clear but the results should be interpreted carefully since the plaque associated spectrum hardly matches the area which was later stained positive with Thioflavin-S. Cases #4a and #4b do not show a plaque associated spectrum after the data processing steps (see also Figure 6). Scale bars: 40 µm.

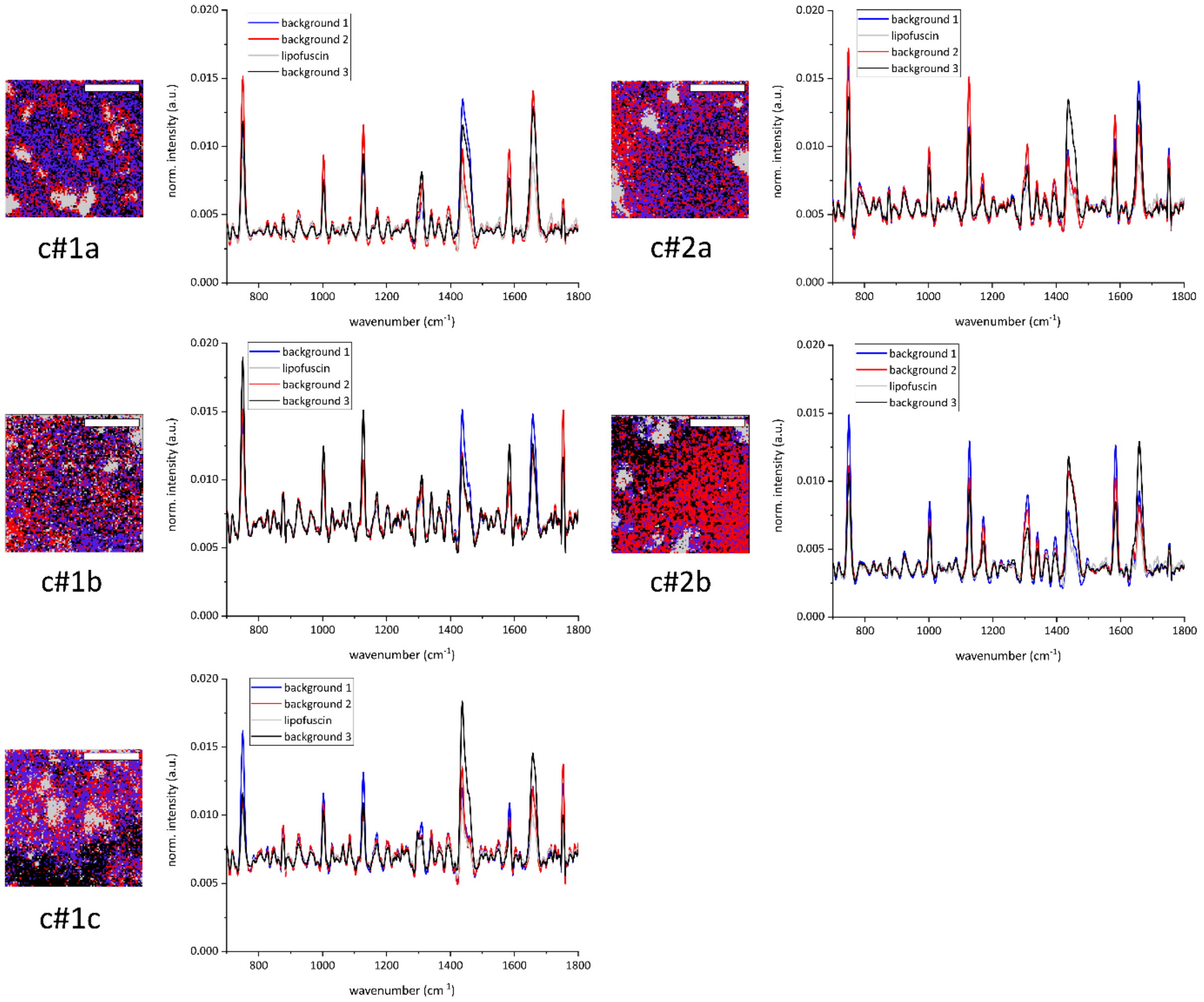

Figure S10: 4-cluster images of all control cases and their corresponding spectra, processed as described for Figure 6. There are intensity variations in each spectrum but no additional peaks are observed. Scale bar: 40 µm.

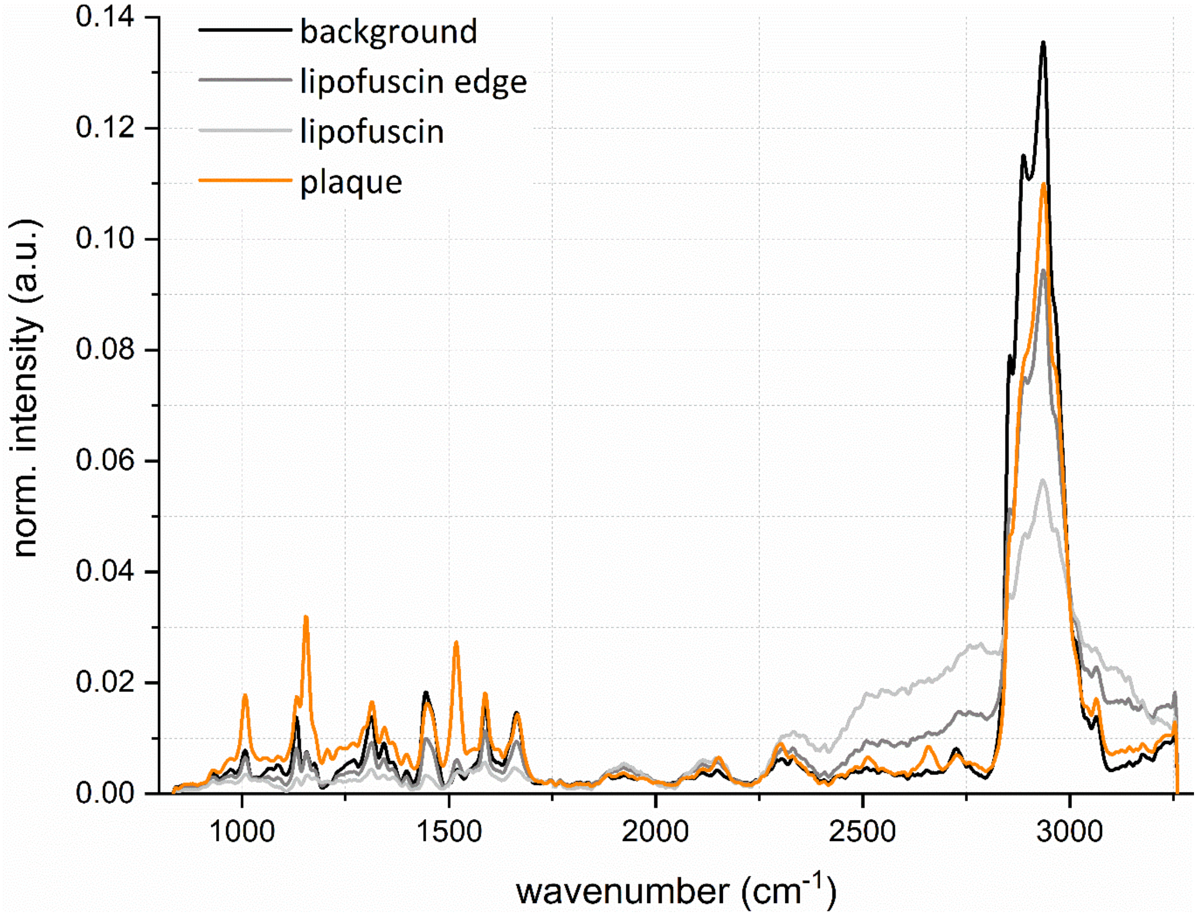

Figure S11: Full recorded Raman spectral range of #1a (as partially shown in Figure 6). In the CH-stretch range we observe, apart from the intensity changes, fewer spectral differences than in the fingerprint range.

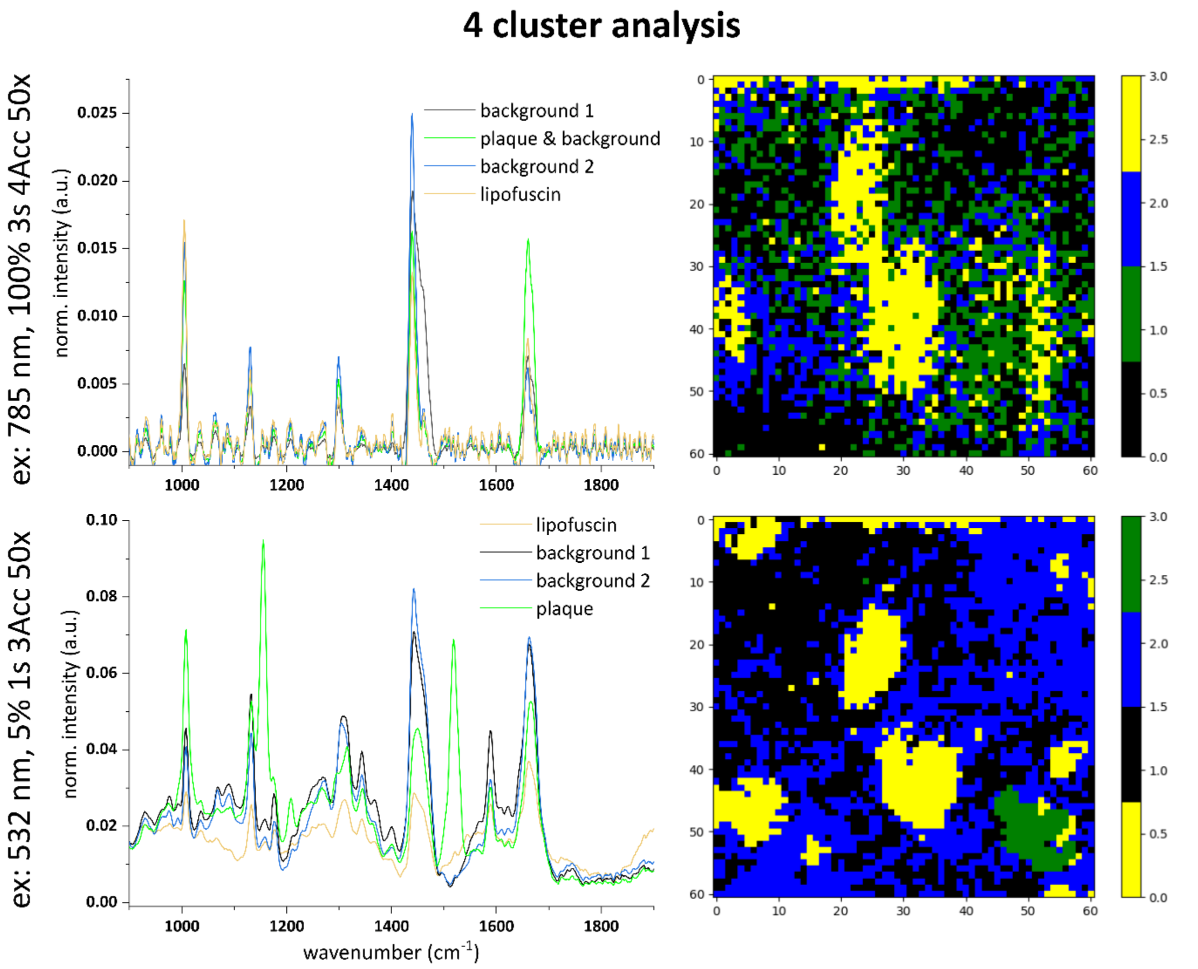

Figure S12 (left): Comparison of the Raman spectra, recorded at the same core plaque location (case #1b, see also Figs. 5 and 6) using 785 nm (top) and 532 nm (bottom) as Raman excitation source. The right column depicts the corresponding 4-cluster images after the raw data were processed in the same manner. The carotenoid associated peaks (around 1150 cm^{-1} and 1520 cm^{-1}) are very prominent in the spectra of the plaque areas with 532-nm excitation (green curve), but are not visible in the spectra obtained with the 785 nm source. Please note that the overall mapping time for the 785 nm source was three times longer. Size of the images is 61 x 61 µm.

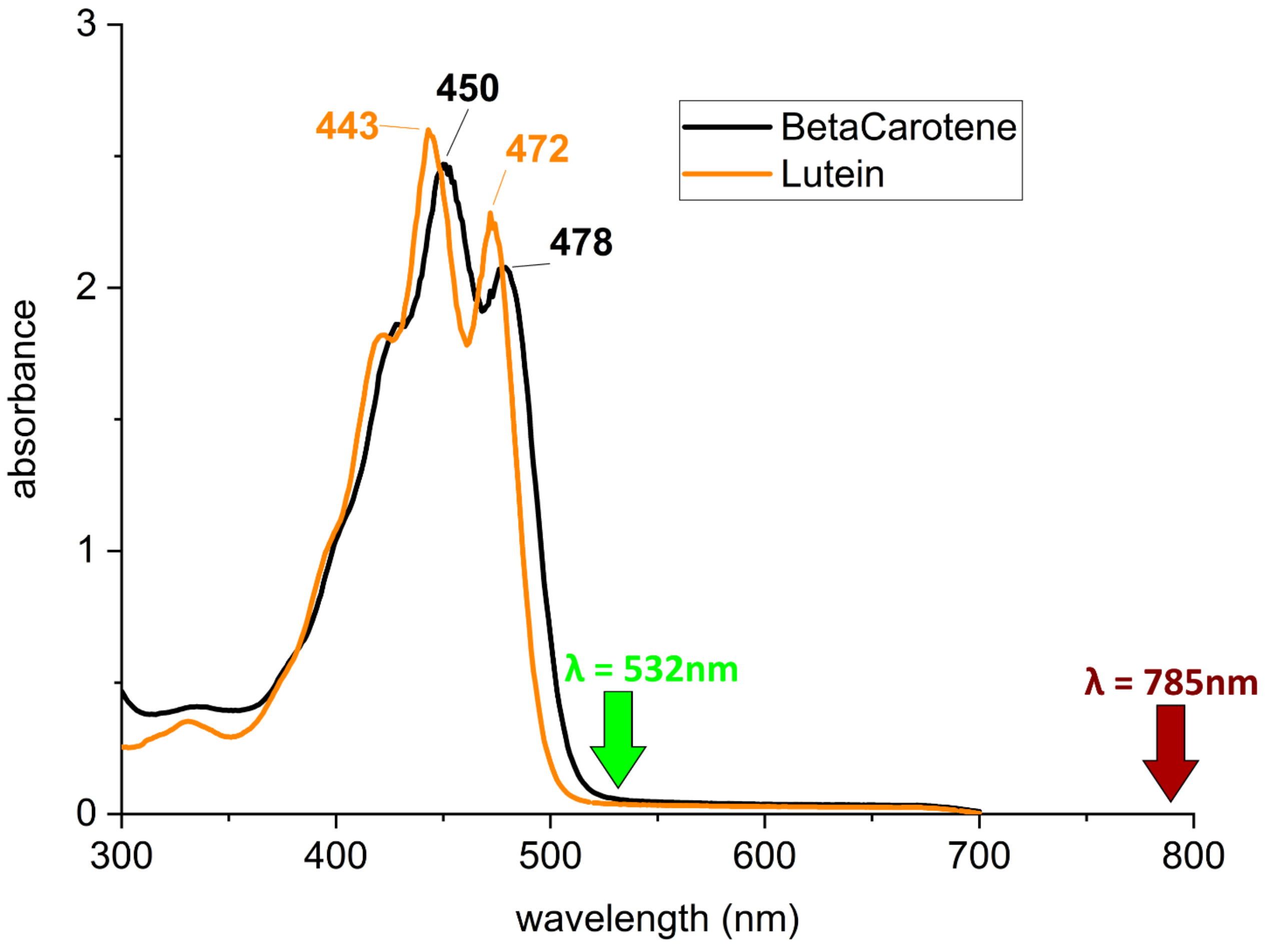

Figure S13: Absorption spectrum of beta-carotene dissolved in hexane, concentration = 1.76 x 10^{-5} M and lutein, concentration = 1.63 x 10^{-5}M. The arrows illustrate the proximity of the green Raman laser to the absorption band (offering pre-resonance enhancement) as opposed to the NIR laser. (Cary 50 single-beam spectrometer; 1 nm spectral bandwidth; 10-mm quartz cuvette; hexane reference).

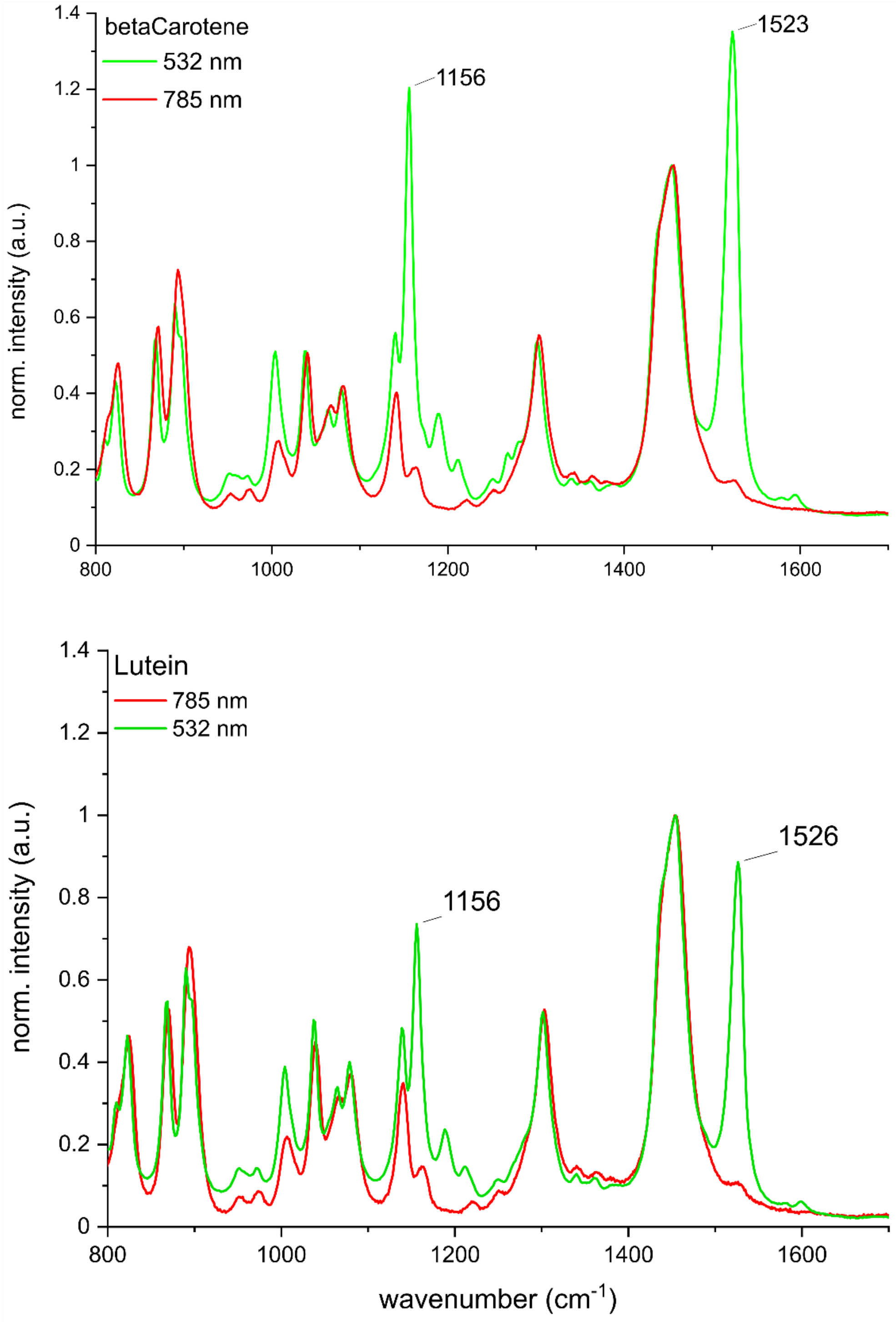

Figure S14: Illustration of the pre-resonance enhancement: Raman spectra of beta-carotene (top) and lutein (bottom) recorded with a 532 nm (green line) and 785 nm (red line) excitation source; $concentration_{betaCarotene} = 1.76 \times 10^{-5}$ M and $concentration_{Lutein} = 1.63 \times 10^{-5}$ M in hexane. The spectra were normalized to the solvent peak at 1455 cm^{-1}, measured in pure hexane (using both excitation sources) and subsequently a baseline has been removed. A pre-resonance enhancement factor of 38 for beta-carotene and of 30 for lutein was determined from the relative intensities of the 1523/6 cm^{-1} peak.

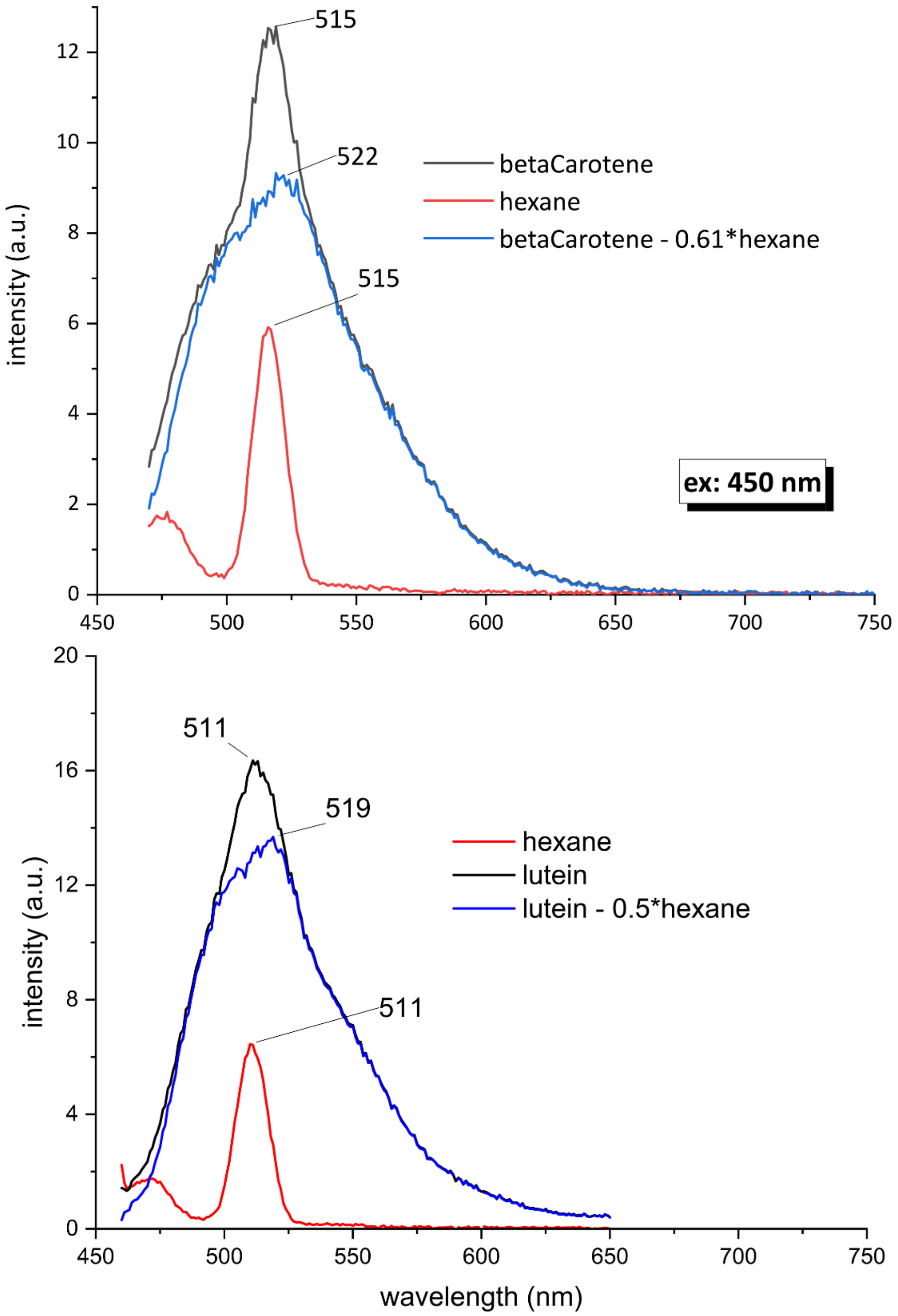

Figure S15: Fluorescence spectra of beta-carotene (top) and lutein (bottom) dissolved in hexane (black line) and of pure hexane (red line) using 450 nm excitation. The blue line shows the pure carotenoid emission spectrum after scaled subtraction of the hexane Raman background. The concentration was 6.2 x10^{-6} M for beta-carotene and 6.9 x 10^{-6} M for lutein. (Cary Eclipse spectrofluorimeter; 10-mm quartz cuvette; exc/em slit widths 10/10 nm).

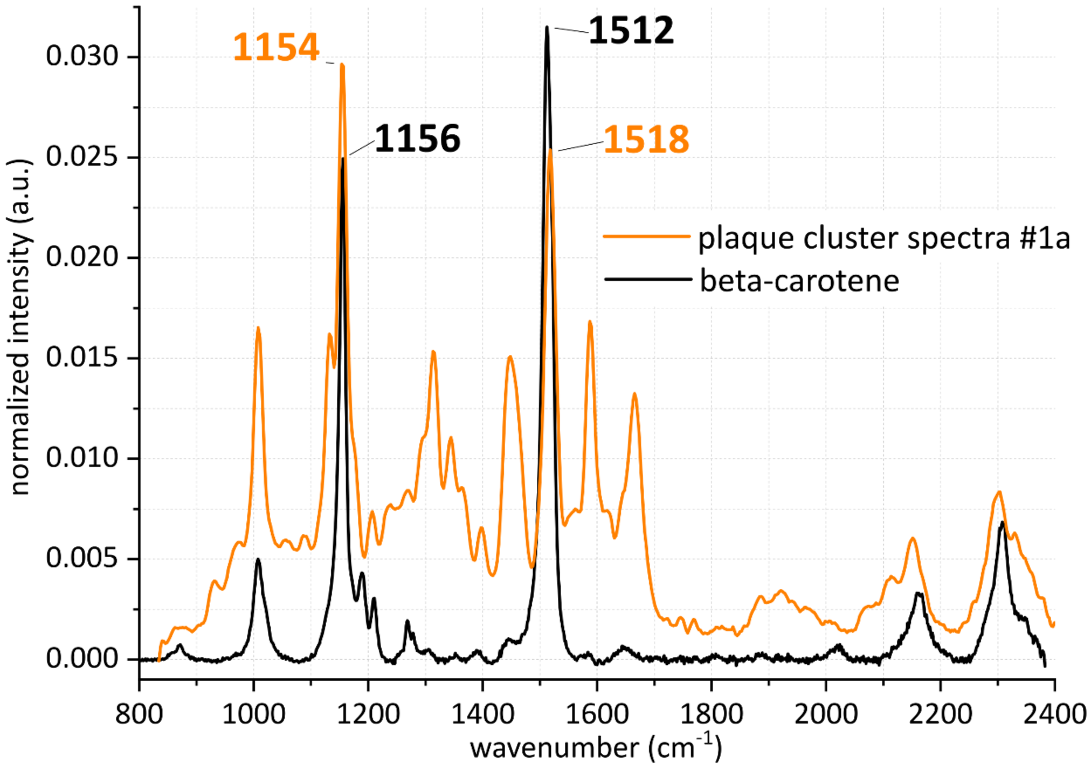

Figure S16: Raman spectral comparison of beta-carotene (6.2 x 10^{-5} M in hexane) with the cluster spectra of plaque #1a, showing a nearly perfect match of the carotenoid peaks with the obtained spectral plaque peaks (around 1007 cm^{-1}, 1150 cm^{-1} and 1520 cm^{-1}). The other peaks in the plaque spectrum also occur in non-plaque areas (see Fig. 6) and correspond to proteins, lipids and other common tissue components.

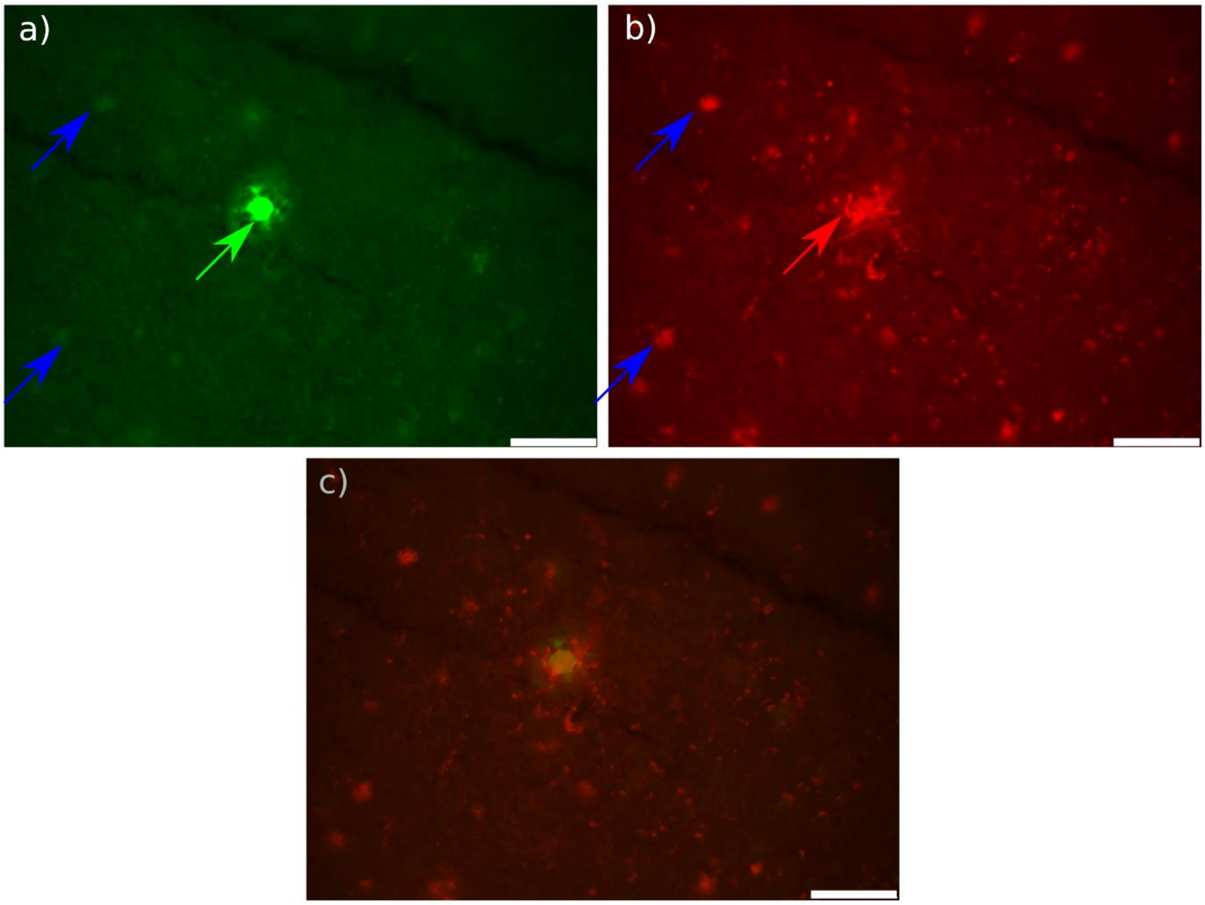

Figure S17: Double staining detection of microglia activity; fluorescence images of an adjacent section of sample #3c. Image a) shows the thio-S positive cored amyloid plaque (green arrow; ex: 480 nm, em: > 512 nm). Image b) shows the tissue stained for microglia (Iba1) activity (red arrow; ex: 560 nm, em: > 593 nm). The blue arrows indicate locations, which are most likely lipofuscin auto-fluorescence. Image c) shows an overlay of images a) and b), highlighting microglia activity next to the cored amyloid deposit. Scale bar: 50 µm

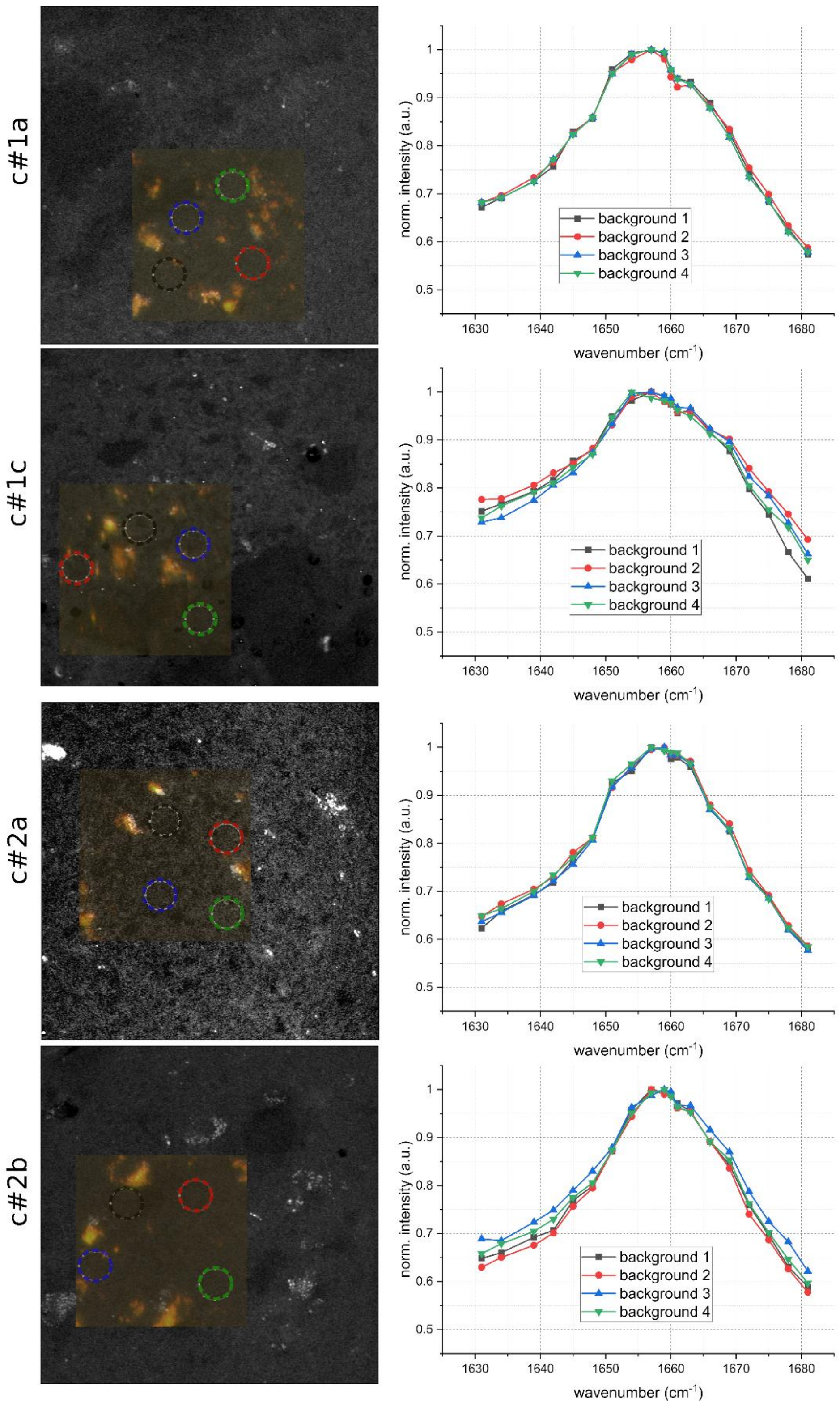

Figure S18: SRS imaging across the protein peak for control cases (no SRS was recorded for c#1b). Random locations were picked (colored circles) and their protein sweep is shown on the right half. As expected, no protein shift was observed. Image size: 204 x 204 µm.
