# Supplementary material for: Multimodal, label-free fluorescence and Raman imaging of amyloid deposits in snap-frozen Alzheimer’s disease human brain tissue
Source: Commun Biol. 2021 Apr 15;4:474. doi: 10.1038/s42003-021-01981-x (PMC8050064; doi:10.1038/s42003-021-01981-x)
Supplement: Supplementary file 3 — Description of Additional Supplementary Files [file 42003_2021_1981_MOESM3_ESM.pdf]

Description of Additional Supplementary Files

File name: Supplementary Data 1

Description: Raw data of Figures 4, 6 and 7.
